# Simvastatin activates single skeletal RyR1 channels but exerts more complex regulation of the cardiac RyR2 isoform

**DOI:** 10.1111/bph.14136

**Published:** 2018-02-05

**Authors:** Elisa Venturi, Chris Lindsay, Sabine Lotteau, Zhaokang Yang, Emma Steer, Katja Witschas, Abigail D Wilson, James R Wickens, Angela J Russell, Derek Steele, Sarah Calaghan, Rebecca Sitsapesan

**Affiliations:** ^1^ Department of Pharmacology University of Oxford Oxford UK; ^2^ School of Biomedical Sciences University of Leeds Leeds UK; ^3^ Department of Chemistry, Chemistry Research Laboratory University of Oxford Oxford UK

## Abstract

**Background and Purpose:**

Statins are amongst the most widely prescribed drugs for those at risk of cardiovascular disease, lowering cholesterol levels by inhibiting 3‐hydroxy‐3‐methylglutaryl (HMG)‐CoA reductase. Although effective at preventing cardiovascular disease, statin use is associated with muscle weakness, myopathies and, occasionally, fatal rhabdomyolysis. As simvastatin, a commonly prescribed statin, promotes Ca^2+^ release from sarcoplasmic reticulum (SR) vesicles, we investigated if simvastatin directly activates skeletal (RyR1) and cardiac (RyR2) ryanodine receptors.

**Experimental Approach:**

RyR1 and RyR2 single‐channel behaviour was investigated after incorporation of sheep cardiac or mouse skeletal SR into planar phospholipid bilayers under voltage‐clamp conditions. LC‐MS was used to monitor the kinetics of interconversion of simvastatin between hydroxy‐acid and lactone forms during these experiments. Cardiac and skeletal myocytes were permeabilised to examine simvastatin modulation of SR Ca^2+^ release.

**Key Results:**

Hydroxy acid simvastatin (active at HMG‐CoA reductase) significantly and reversibly increased RyR1 open probability (Po) and shifted the distribution of Ca^2+^ spark frequency towards higher values in skeletal fibres. In contrast, simvastatin reduced RyR2 Po and shifted the distribution of spark frequency towards lower values in ventricular cardiomyocytes. The lactone pro‐drug form of simvastatin (inactive at HMG‐CoA reductase) also activated RyR1, suggesting that the HMG‐CoA inhibitor pharmacophore was not responsible for RyR1 activation.

**Conclusion and Implications:**

Simvastatin interacts with RyR1 to increase SR Ca^2+^ release and thus may contribute to its reported adverse effects on skeletal muscle. The ability of low concentrations of simvastatin to reduce RyR2 Po may also protect against Ca^2+^‐dependent arrhythmias and sudden cardiac death.

**Abbreviations**
AF: atrial fibrillation
AICAR: 5‐aminoimidazole‐4‐carboxamide ribonucleotide
CCD: central core disease
FDB: flexor digitorum brevis
HMG‐CoA: 3‐hydroxy‐3‐methylglutaryl CoA
Log D: partition coefficient
MH: malignant hyperthermia
Po: open probability
RyR: ryanodine receptor
Sim‐H: simvastatin hydroxy acid
Sim‐L: simvastatin lactone
SR: sarcoplasmic reticulum
*Trans*: luminal

## Introduction

Statins are competitive inhibitors of **3‐hydroxy‐3‐methylglutaryl‐CoA (HMG‐CoA) reductase**, the rate‐limiting enzyme in the synthesis of cholesterol. They are the most widely prescribed medication for the treatment of hypercholesterolaemia and prevention of cardiovascular disease. Furthermore, statin use is increasing with recent reductions in the cardiovascular risk threshold for statin prescription (NICE, 2014). The efficacy of statin drugs has been demonstrated in many large clinical trials (Collins *et al.,* 2016), and while their success has been unrivalled, they are also associated with causing a variety of muscle‐related complaints (Bruckert *et al.,* 2005; Buettner *et al.,* 2012; Stroes *et al.,* 2015). Patients prescribed statin treatment often report muscle pain and weakness, and in serious cases, fatal rhabdomyolysis can occur (Hodel, 2002). Observational studies have suggested that between 10 and 15% of patients undergoing statin therapy experience these side effects, and this has restricted their use in certain contexts. As a result many patients have so far been unable to benefit from statins protection against cardiovascular disease, as well as their pleiotropic cardioprotective effects (Thompson *et al.,* 2003).

All statins are reported to cause muscle‐related side effects; however, the occurrence of these adverse effects is not correlated with potency of HMG‐CoA reductase inhibition but does appear to become more severe with increasing doses (Armitage, 2007). The mechanisms underlying this toxicity have not yet been fully elucidated although there are several suggested off‐target mechanisms such as inhibition of mitochondrial respiration (Schirris *et al.,* 2015) and induction of apoptosis (Johnson *et al.,* 2004; Borahay *et al.,* 2014). It has also been reported that certain statins can stimulate the release of Ca^2+^ from isolated sarcoplasmic reticulum (SR) vesicles (Inoue *et al.,* 2003) and from the SR in muscle fibres (Sirvent *et al.,* 2005a; Sirvent *et al.,* 2005b). It is therefore possible that statins directly affect the function of the ryanodine receptor **RyR1**, the predominant SR Ca^2+^ release channel in skeletal muscle. Mutations to RyR1 result in a range of serious skeletal muscle diseases including congenital myopathies such as malignant hyperthermia (MH), central core disease (CCD) and exercise‐induced rhabdomyolysis (Sambuughin *et al.,* 2005; Chelu *et al.,* 2006). In MH and CCD, muscle weakness and hyperthermia are associated with RyR1 channels that display an enhanced sensitivity to agonists (Chelu *et al.,* 2006). Interestingly, recent population studies have reported a link between statin‐induced myopathy and patients with RyR1 mutations that give rise to MH (Zutt *et al.,* 2014; Hedenmalm *et al.,* 2015). In addition, it has been shown that mice carrying the MH‐linked mutation, Y524S, are more sensitive to **simvastatin** than wild‐type animals, demonstrating increased SR Ca^2+^ leak from flexor digitorum brevis (FDB) fibres, as well as increased heat production and muscle contractures (Knoblauch *et al.,* 2013).

In view of the known association of ‘leaky’ RyR1 channels with severe muscle myopathies, we therefore investigated if simvastatin exerts a direct effect on the gating and conducting properties of the native skeletal RyR1 channel complex incorporated into planar phospholipid bilayers under voltage‐clamp conditions. We also examined if simvastatin could alter the spontaneous SR Ca^2+^ release from RyR clusters (Ca^2+^ sparks) *in situ* in single isolated, permeabilised rat skeletal muscle cells.

There are three mammalian isoforms of RyR. RyR1 is found predominately in skeletal muscle, **RyR2** in cardiac muscle and RyR3 is widely expressed in various tissues but often at low levels (Zucchi and Ronca‐Testoni, 1997). Although a few agents have been suggested to specifically interact with only one of these mammalian isoforms, a ligand that modulates the function of one RyR isoform will usually interact with other isoforms even if the response is subtly different (Venturi *et al.,* 2014). Therefore, since statins are given to patients with a history of heart disease and an increased likelihood of experiencing arrhythmias, it is important to investigate whether statins also affect cardiac RyR2 channel function. Altering the single‐channel conductance or open probability (Po) of RyR2 might influence the release of SR Ca^2+^ thereby potentially affecting contractility and the propensity for Ca^2+^‐dependent arrhythmias. Consequently, we have also investigated whether simvastatin alters the single channel behaviour of RyR2 incorporated into bilayers and Ca^2+^ sparks in isolated, permeabilised ventricular cardiomyocytes.

**Figure 1:**
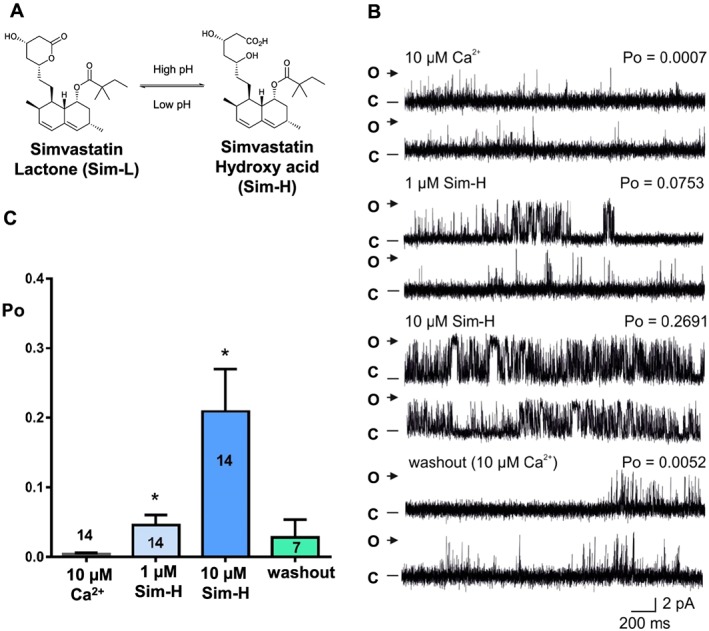
The effects of cytosolic Sim‐H on RyR1 channel gating. (A) The chemical structures of Sim‐L and Sim‐H. The interconversion between lactone and hydroxy acid forms shows a strong dependence on pH. (B) Representative mouse skeletal RyR1 single‐channel current fluctuations in the presence of 10 μM cytosolic Ca^2+^ alone (top trace), after cytosolic addition of 1 and 10 μM Sim‐H (as indicated) and after wash‐out of Sim‐H back to control conditions (10 μM cytosolic Ca^2+^, bottom trace). The Po above each trace refers to the value determined over 3 min. O and C indicate the open and closed channel levels respectively. (C) Mean RyR1 Po values with 10 μM cytosolic Ca^2+^ as sole channel activator and after addition of 1 μM Sim‐H, 10 μM Sim‐H and wash‐out of Sim‐H. Mean and SEM are shown; numbers on the bars indicate number of independent experiments [*n* = 14 (10 μM Ca^2+^_,_ 1 μM and 10 μM Sim‐H), *n* = 7 (washout); *P < 0.05].

Simvastatin is one of the most commonly prescribed statins. While there are a variety of structurally distinct statin molecules, all have in common a modified hydroxyglutaric acid unit which is responsible for inhibition of HMG‐CoA reductase (Istvan and Deisenhofer, 2001). This unit may exist as either a lipophilic lactone ring (to give the closed form of simvastatin lactone, Sim‐L) or an open form as a more hydrophilic free acid (simvastatin hydroxy acid, Sim‐H). Simvastatin is prescribed in its lactone form, which is subsequently converted *in vivo* to the open active form (Figure 1A) (Kearney *et al.,* 1993). This lipophilicity (log D at pH 7.4) (McKenney, 2003) is also correlated with the relative occurrence of muscle‐related side effects; cerivastatin (log D ~ 1.75), which was withdrawn from the market in 2001, is also the most lipophilic statin that has been used clinically (White, 2002). More lipophilic drugs typically display greater levels of passive diffusion across cellular membranes, thus affecting the distribution of the drug. For example, simvastatin (log D ~ 1.60 in Sim‐L form) can easily cross the blood–brain barrier, while the structurally similar but more hydrophilic pravastatin (log D ~ −0.84 in lactone form) does not (Hamelin and Turgeon, 1998; McKenney, 2003).

Our results show RyR isoform‐specific effects of simvastatin. We demonstrated that ≥1 μM Sim‐H significantly increases the Po of RyR1 in a cytosolic Ca^2+^‐dependent manner. In line with these experiments, statin treatment tends to increase the frequency of Ca^2+^ sparks in permeabilised skeletal muscle cells. In contrast, low concentrations of Sim‐H (1 μM) reduced the Po of RyR2. Sim‐H also tended to reduce the frequency of Ca^2+^ sparks in isolated permeabilised ventricular cardiomyocytes. It will be important to investigate the effects of other prescribed statins on cardiac and skeletal RyR channel function as the effects described in this paper may contribute to the unwanted muscular side effects or to, as yet, uncharacterized effects on cardiac function.

## Methods

### Animals and ethical approval

Animal studies are reported in compliance with the ARRIVE guidelines (Kilkenny *et al*., 2010; McGrath and Lilley, 2015). Mouse skeletal muscle was used for RyR1 single‐channel experiments and [^3^H]‐ryanodine binding; sheep hearts were used for RyR2 single‐channel experiments and [^3^H]‐ryanodine binding; rat skeletal fibres and cardiac myocytes were used for analysis of Ca^2+^ sparks in permeabilised cells. All rodent work was carried out in accordance with the Directive 2010/63/EU of the European Parliament with local approval of the Animal Research Committee according to the regulations on animal experimentation at the University of Oxford (mouse) and the Animal Welfare and Ethical Review board at the University of Leeds (rat). Rats were housed in a conventional unit, in filter top cages with Aspen bedding on a 12 h light/dark cycle with free access to food and water. Mice were housed in a specific pathogen free unit in individually ventilated cage on a 12 h light/dark cycle with free access to food and water. Sheep hearts were obtained from an abattoir. To comply with the ‘three Rs’ principles, we prepared SR membranes from animals that were killed for other purposes wherever possible (sheep hearts, mouse skeletal muscle). Our choice of tissue was based on the similarity of RyR proteins between species and designed to minimize animal use without compromising the integrity of the data. Identity between mouse (E9PZQ0.1) and rat (F1LMY4.1) RyR1 sequences is 96%. RyR2 sequences are also 96% identical between sheep (XP_014959831.1) and rat (B0LPN4).

### Single‐channel recordings

Heavy sarcoplasmic reticulum (HSR) membrane vesicles were prepared from mouse skeletal muscle or sheep hearts as described previously (Sitsapesan and Williams, 1990). Mice (C57BL/6) were 10–14 weeks old, of either sex. Sheep (Suffolk breed) were 8–10 months old, of either sex.

RyR1 or RyR2 channels were incorporated into planar phospholipid bilayers as previously described (Sitsapesan *et al.,* 1991), and current fluctuations through RyR channels were recorded under voltage‐clamp conditions with 250 mM HEPES, 80 mM Tris, 10 μM free Ca^2+^, pH 7.2, on the cytoplasmic side and 250 mM glutamic acid, 10 mM HEPES, pH to 7.2 with Ca(OH)_2_ (free [Ca^2+^] approximately 50 mM) on the *trans* (luminal) side of the bilayer at 21°C. The *trans* chamber was voltage‐clamped at ground. The compound to be investigated was added to the cytosolic chamber. The free [Ca^2+^] and pH of the solutions were maintained constant during the experiment and were determined using a Ca^2+^ electrode (Orion 93‐20, Thermo Fisher Scientific, UK) and a Ross‐type pH electrode (Orion 81‐55, Thermo Fisher Scientific, UK) as previously described (Sitsapesan *et al.,* 1991). Sub‐activating cytosolic Ca^2+^ levels were obtained by addition of 1 mM EGTA to the cytosolic chamber and the approximate free [Ca^2+^] calculated using the MaxChelator programme (http://maxchelator.stanford.edu). The addition of Sim‐H or Sim‐L did not alter the free [Ca^2+^] or pH of solutions.

### Single‐channel analysis

Single‐channel recordings were digitized at 20 kHz and low‐pass filtered at 800 Hz. Po was determined over 3 min of continuous recording at 0 mV using 50% threshold analysis (Colquhoun and Sigworth, 1995) in Clampfit 10.6 (Molecular Devices, USA) as previously described (Sitsapesan and Williams, 1990; Sitsapesan *et al.,* 1991). Where Po values are shown in figures, the Po above each trace refers to the value determined over 3 min for that particular channel. Where more than one channel was incorporated into the bilayer, Po is reported as an average (total Po divided by number of channels). Lifetime distributions were constructed using Clampfit 10.6 (Molecular Devices, USA) and fitted to a probability density function by the method of maximum likelihood (Colquhoun and Sigworth, 1995) according to the following equation:

$$f\left(t\right)=\sum_{i}^{N}a_{i}f_{0}\left(t-\ln\tau_{i}\right)$$

where i is the number of exponential components of the distribution, τ_i_ are the time constants and a_i_ are the fractions of the total events represented by the *i*th component. The set of parameters were adjusted by the maximum likelihood iterative algorithm until the optimum value of the log of likelihood L was reached (Blatz and Magleby, 1986).

### [^3^H]‐ryanodine binding

The binding of [^3^H]‐ryanodine to RyR was measured as previously described (Sigalas *et al.,* 2009). In brief, mouse skeletal mixed membrane vesicles containing 500 μg protein·mL^−1^, or sheep cardiac SR vesicles containing 130 μg protein·mL^−1^ were incubated at 37°C for 90 min with constant shaking in buffer consisting of 250 mM HEPES, 80 mM Tris, 10 μM free Ca^2+^, pH 7.2 in the presence of 5 nM [^3^H]‐ryanodine. Nonspecific binding was determined in the presence of 1000‐fold excess unlabelled **ryanodine**. The bound and free ligand were separated by rapid filtration through Whatman GF/B glass microfibre filters. The [^3^H]‐ryanodine retained in the filters was quantified by liquid scintillation spectrometry using a scintillation counter.

### Cellular Ca^2+^‐ release

Collagenase‐digested flexor digitorum brevis (FDB) fibres (Pickering *et al.,* 2009) and ventricular myocytes (Calaghan *et al.,* 1998) isolated from male 4–6 week old Wistar rats (100–150 g) were used for confocal microscopy. FDB fibres were permeabilised by 2 min exposure to 0.005% saponin and bathed in intracellular solution containing (mM): K_2_SO_4_ 95, HEPES 10, EGTA 0.47, MgCl_2_ 6, Na_2_‐ATP 5, Na_2_‐creatine phosphate 10, glucose 10, CaCl_2_ 0.13, pH 7.2. Ventricular myocytes were permeabilised by 10 min exposure to 0.001% saponin and bathed in intracellular solution containing (mM): KCl 100, HEPES 25, EGTA 0.35, MgCl_2_ 5.72, Na_2_ATP 5, Na_2_‐creatine phosphate 10, CaCl_2_ 0.05, pH 7. Permeabilised FDB fibres and ventricular myocytes were loaded with Fluo‐3 pentapotassium salt (25 or 10 μM respectively) and perfused with 10 μM Sim‐H for 5 min. Confocal images were acquired with a Nikon Eclipse TE300 inverted microscope equipped with a confocal scanhead, Bio‐Rad MicroRadiance 2000 and a ×60 water‐immersion objective. Fluo‐3 pentapotassium was excited with the 488 nm line of a 20 mW coherent sapphire laser, and the fluorescence emitted was measured at >515 nm. Images were acquired in line scan mode (every 6 ms). Ca^2+^ sparks were identified and analysed with ImageJ software (NIH) using the Sparkmaster plugin (Picht *et al.,* 2007). Events with a full width at half maximum <1 μm were filtered from the data.

### LC‐MS analysis

Sim‐H and Sim‐L were prepared as 10 mM solutions in water and DMSO, respectively, and stored at −78°C, and warmed to room temperature immediately prior to injection; 1 μL of each solution was added to 1 mL of water or Tris/HEPES (250 mM HEPES, 80 mM Tris, 10 μM free Ca^2+^, pH 7.2) buffer, respectively, and sampling by LC‐MS was begun immediately. Samples were kept at room temperature for the duration of the analysis. Analyses were performed using a Thermo Exactive mass spectrometer equipped with Waters Acquity liquid chromatography system. Instrument control was performed using Thermo Xcalibur Software. Electrospray source conditions were adjusted to maximize sensitivity; 3 μL of each sample was injected onto the system. The column was a 2.1 × 50 mm (3 μm) ACE equivalence C18. A mixed eluent gradient was employed and is described in Supporting Information Table S3. The four sample types were analysed sequentially; the 5 min analysis time for each sample provided a 20 min sampling frequency for each sample. Mass analysis was performed using MestReNova MS software (2016, V11.0), where the peaks were integrated to determine relative percentages of Sim‐L and Sim‐H over time.

### Statistical analysis

The data and statistical analysis comply with the recommendations on experimental design and analysis in pharmacology (Curtis *et al.,* 2015) and were carried out using GraphPad Prism 7. Normality was checked using the D'agostino and Pearson test. Where *n* ≥ 5, normally distributed data are expressed as mean ± SEM and statistical comparisons were made using Student's *t*‐test. Where multiple treatments were compared, ANOVA followed by a modified *t*‐test was used to assess the difference between treatments (Galfre *et al.,* 2012). Data that were not normally distributed are expressed as median + interquartile range; statistical comparisons were made using the Mann–Whitney test. A *P* value of <0.05 was taken as significant. Variations in *n* numbers for single‐channel experiments were due to bilayers breaking during the course of the experiment, which precluded further measurements being taken. In all cases, where skeletal and cardiac SR was used, data were obtained from at least five different membrane preparations prepared from five or more animals. For permeabilised skeletal and cardiac cell experiments, spark parameters were obtained from ≥66 cells from five rats.

### Materials

Simvastatin sodium salt (Sim‐H) was purchased from CalBioTech (567021). Simvastatin lactone (Sim‐L) was purchased from Sigma‐Aldrich (Dorset, UK). All other chemicals were purchased from Sigma‐Aldrich (Dorset, UK) or VWR (Poole, UK) unless stated otherwise. Water was deionized (Millipore, Harrow, UK), and all solutions used in single‐channel experiments were filtered through a membrane with a 0.45 μm diameter pore (Millipore, Harrow, UK).

### Nomenclature of targets and ligands

Key protein targets and ligands in this article are hyperlinked to corresponding entries in http://www.guidetopharmacology.org, the common portal for data from the IUPHAR/BPS Guide to PHARMACOLOGY (Harding *et al.,* 2018), and are permanently archived in the Concise Guide to PHARMACOLOGY 2017/18 (Alexander *et al*., 2017a,b).

## Results

### Simvastatin directly modulates RyR1 and RyR2 channel function

**Figure 2:**
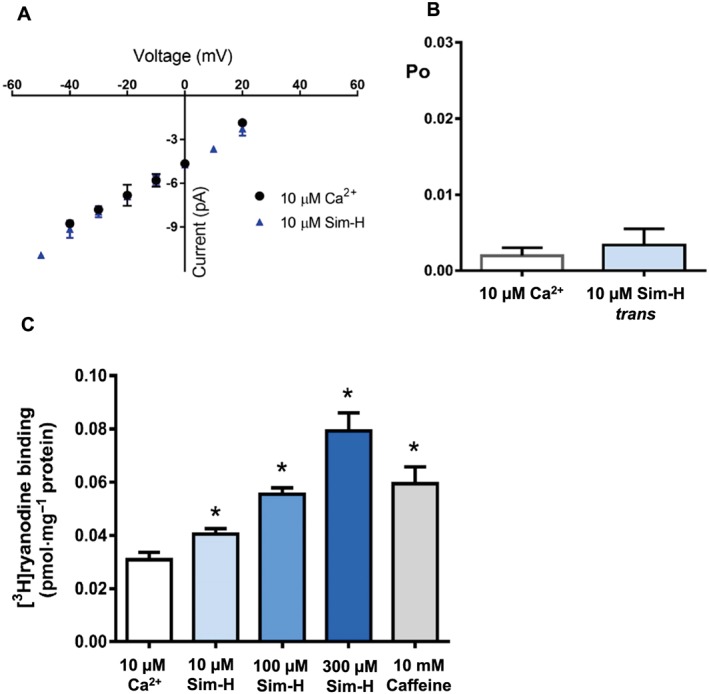
The effects of Sim‐H on RyR1 channel function. (A) Single‐channel current–voltage relationship for RyR1 before and after simvastatin treatment. (B) Mean RyR1 Po values before and after addition of 10 μM Sim‐H to the luminal (*trans*) side of the bilayer (mean and SEM are shown; *n* = 6). (C) Stimulation of [^3^H]‐ryanodine binding to mouse skeletal muscle SR membrane vesicles by 10 μM cytosolic Ca^2+^ alone (control), and in the additional presence of Sim‐H (at indicated concentrations) or caffeine (10 mM) (mean and SEM; *n* = 5; *P < 0.05).

The effects of the hydroxy acid form of simvastatin (Sim‐H; see Figure 1A) on native skeletal RyR1 and cardiac RyR2 channel function were investigated first as this is the form which binds to HMG‐CoA reductase. In the presence of 10 μM cytosolic‐free Ca^2+^, cytosolic addition of Sim‐H caused a concentration‐dependent increase in the Po of RyR1 (Figure 1B, C). The increase in RyR1 Po was readily reversed to control values after washout of the cytosolic chamber. Sim‐H did not affect single‐channel conductance (Figure 2A) under these conditions nor were significant changes in channel Po observed when Sim‐H was added to the luminal side of the bilayer (Figure 2B). Very high concentrations of simvastatin were not used in single‐channel experiments because statins are lipophilic agents that would be expected to destabilize artificial bilayers. To demonstrate that the effects of simvastatin are not artefacts of the artificial bilayer membrane, the binding of [^3^H]‐ryanodine to isolated skeletal SR vesicles was also assessed. Ryanodine only binds to open RyR channels, thus providing an index of Po for populations of RyR in their native membranes. This is an approximate Po assay because it depends on ryanodine binding to RyR, and the conditions of the binding assay can never be exactly equivalent to those of a single‐channel experiment. Sim‐H stimulated the binding of [^3^H]‐ryanodine at μM concentrations (10–300 μM).

Figure 2C compares the effect of an optimal concentration of caffeine suggesting that Sim‐H is more potent and more effective as an activator of RyR1 than caffeine (Figure 2C).

**Figure 3:**
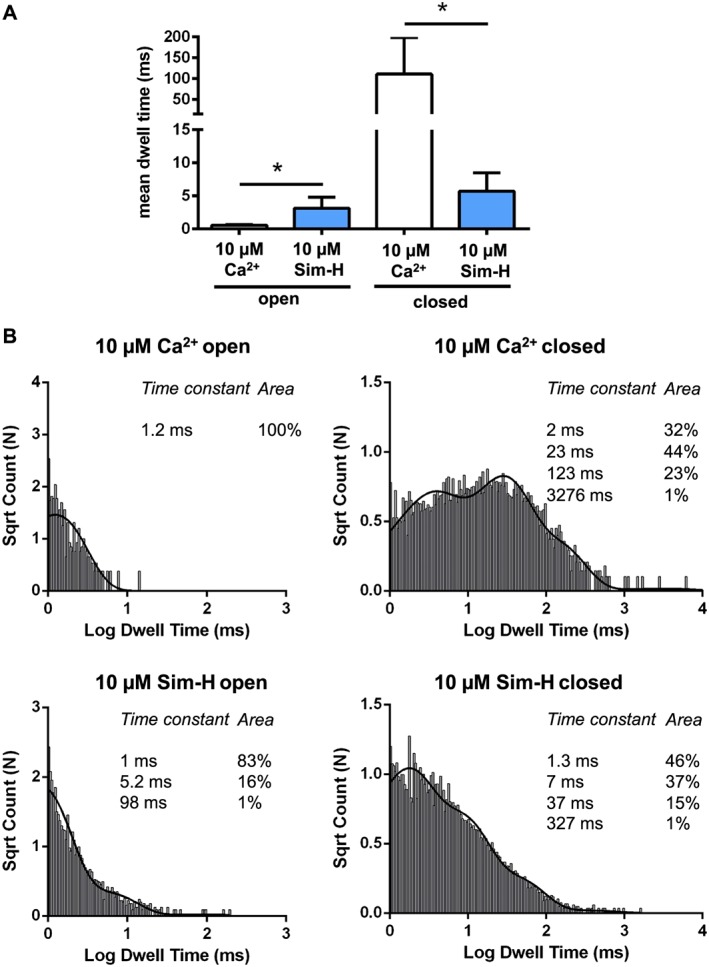
Effects of cytosolic Sim‐H on lifetime distributions. (A) The effect of Sim‐H on mean open and closed times (*P < 0.05; mean and SEM, *n* = 5). (B) Open and closed lifetime distributions and probability density functions for a representative RyR1 channel activated solely by 10 μM cytosolic Ca^2+^ (top), and after addition of Sim‐H (bottom). The τ values (time constants) and percentage areas are shown.

Where only one channel had incorporated into the bilayer, we performed lifetime analysis to examine the mechanism of the statin‐induced increase in Po. Figure 3A shows that there was a small increase in the mean open time but a 20‐fold reduction in the mean closed time. Detailed analysis of the open and closed lifetime distributions (see typical example in Figure 3B and see Supporting Information Table S1 for τ values and percentage areas of replicates) identified additional longer open states and demonstrated that all closed lifetime constants were markedly shortened. Thus, although Sim‐H caused a small increase in open dwell times, the major mechanism for the increase in RyR1 Po was an increase in frequency of channel opening.

**Figure 4:**
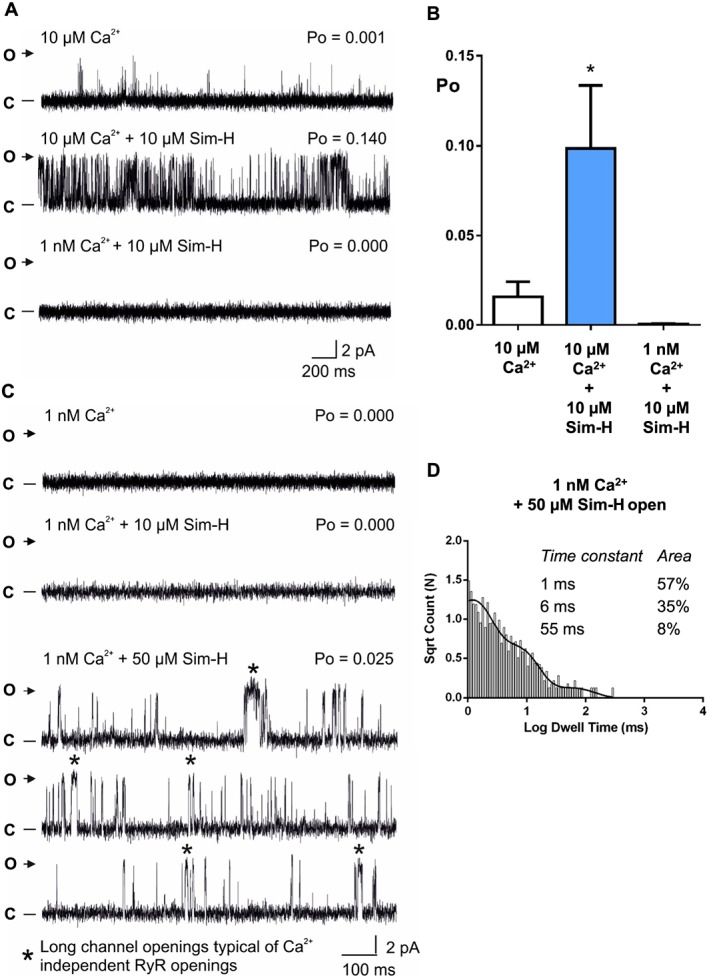
Ca^2+^‐dependent and ‐independent actions of Sim‐H on RyR1 channel gating. (A) Representative RyR1 channel recordings in the presence of 10 μM cytosolic Ca^2+^ (top trace), and after sequential additions of 10 μM cytosolic Sim‐H (second trace), and 1 mM EGTA (free cytosolic [Ca^2+^] ~1 nM) (bottom trace). The Po above each trace refers to the value determined over 3 min. O and C indicate the open and closed channel levels respectively. (B) Mean data and SEM from six independent experiments similar to the one shown in (A); *n* = 6; *P < 0.05. (C) Representative RyR1 single‐channel behaviour in the presence of subactivating cytosolic Ca^2+^ (free [Ca^2+^] ~1 nM) (top trace) to show zero openings. There are still no (zero) openings after addition of 10 μM Sim‐H (second trace). The lower traces provide examples of the longer type of openings elicited after addition of 50 μM Sim‐H. O and C indicate the open and closed channel levels respectively. The symbol (*) highlights long channel openings typical of Ca^2+^‐independent RyR openings (the nature of these openings has been described previously; Sitsapesan and Williams, 1990, Kermode *et al.,* 1998). (D) Open lifetime distributions and probability density function for a representative RyR1 channel activated solely by 50 μM Sim‐H in the absence of activating cytosolic Ca^2+^ [~1 nM].

To further investigate the mechanisms underlying Sim‐H activation of RyR1, we lowered the free cytosolic [Ca^2+^] to sub‐activating levels (~1 nM) after channel activation by 10 μM Sim‐H. A typical experiment is shown in Figure 4A. In the presence of activating [Ca^2+^], the response to 10 μM Sim‐H can be observed. Subsequent lowering of the cytosolic [Ca^2+^] to sub‐activating concentrations (~1 nM; third trace) completely abolished any channel openings (Po = 0). Thus, the 10 μM Sim‐H is absolutely dependent on the presence of activating levels of cytosolic Ca^2+^ in order to raise RyR1 Po. The mean data are shown in Figure 4B. Since cytosolic Ca^2+^ (like Sim‐H) also activates RyR1 predominantly by increasing frequency of opening (Smith *et al.,* 1986), it appears that Sim‐H (at low concentrations) is sensitizing the channel to cytosolic Ca^2+^. However, channel openings can be induced in a Ca^2+^‐independent manner if the concentration of Sim‐H is increased. In Figure 4C, inspection of the trace shows the longer type of openings that are characteristic of the RyR Ca^2+^‐independent type openings that have previously been described for ligands such as caffeine and ATP (Sitsapesan and Williams, 1990; Kermode *et al.,* 1998). Figure 4D confirms the effect on open times (see also supporting information Table S2). Hence, Sim‐H can activate RyR1 by sensitizing the channel to cytosolic Ca^2+^ at low concentrations, but higher concentrations can activate the channel in a Ca^2+^‐independent manner.

**Figure 5:**
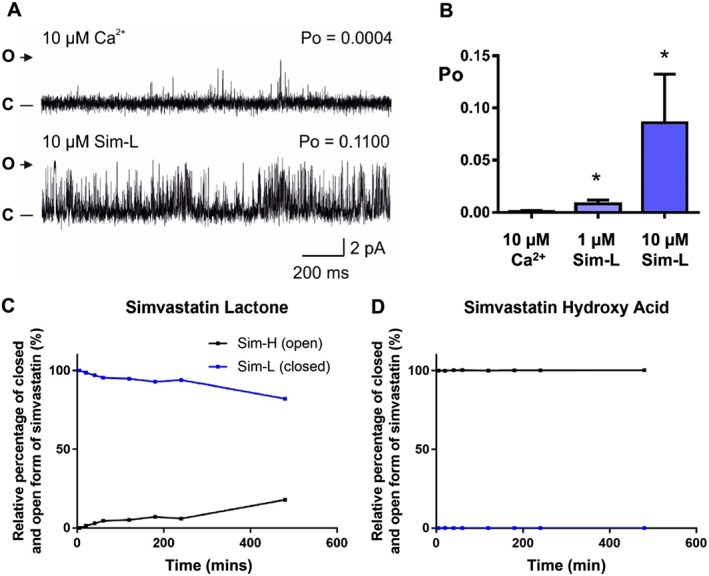
The effects of Sim‐L on RyR1 channel gating. (A) A representative RyR1 channel recording in the presence of 10 μM cytosolic Ca^2+^ alone (top trace) and after addition of 10 μM cytosolic Sim‐L (bottom trace) is shown. The Po above each trace refers to the value determined over 3 min. O and C indicate the open and closed channel levels respectively. (B) Mean RyR1 Po values in the presence of 10 μM Ca^2+^ alone and after addition of 1 μM Sim‐L and 10 μM Sim‐L (mean and SEM; *n* = 6; * *P* < 0.05). (C) and (D) show interconversion of Sim‐L and Sim‐H in the buffer used for the single‐channel experiments (Tris/HEPES) over time (mean of three independent experiments). The conversion of Sim‐L to Sim‐H is <2% over the time course of the single channel experiment (<20 min).

To investigate the possible site of interaction of the simvastatin molecule with RyR1, we examined the effect of the ‘HMG‐CoA inactive’ lactone form (Sim‐L) on RyR1 channel gating. Sim‐L also significantly activated RyR1 channels (Figure 5A, B), suggesting that the region crucial for binding to and inhibiting the HMG‐CoA reductase is different from the region that is most important for interacting with and modulating RyR1 activity.

It has been reported previously that the interconversion of statins has a strong pH dependence (Taha *et al.,* 2016), so to confirm that the lactone form (Sim‐L) does not undergo hydrolysis during the time frame of our single‐channel experiments, we measured the interconversion rate of the lactone form (Sim‐L) into the open ring form of simvastatin (Sim‐H) using LC‐MS (Figure 5C). We did the same for Sim‐H (Figure 5D). Sim‐L and Sim‐H were each dissolved in the buffer used for the single‐channel experiments (Tris/HEPES). In the case of Sim‐L, we found that even after 1 h, 95% of the simvastatin detected was still in the closed‐ring lactone form. Hence, in the experiment shown in Figure 5A, at least 99% of Sim‐L would remain by the end of the experiment. For Sim‐H, there was no apparent tendency for free acid to undergo ring‐closing to form Sim‐L, as no detectable levels of Sim‐L were found even after 1 h incubation (Figure 5D).

**Figure 6:**
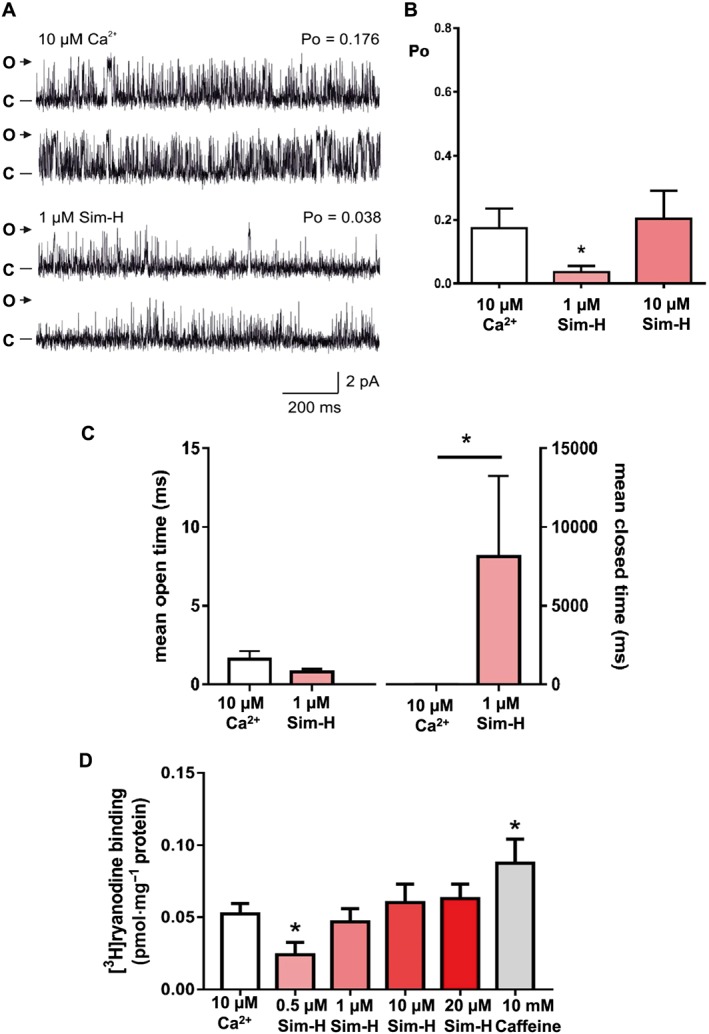
The effects of Sim‐H on sheep cardiac RyR2 function. (A) A typical recording of RyR2 in the presence of 10 μM cytosolic Ca^2+^ (top two traces) and after addition of 1 μM Sim‐H (bottom two traces) is shown. The Po above each trace refers to the value determined over 3 min. O and C indicate the open and closed channel levels respectively. (B) Mean and SEM RyR2 Po values in the presence of 10 μM cytosolic Ca^2+^ as the sole channel activator and in the added presence of increasing concentrations of Sim‐H (*n* = 7; *P < 0.05). (C) The effects of Sim‐H on RyR2 mean open and closed lifetimes. Results are presented as mean and SEM, *n* = 7. *P < 0.05 before and after addition of 1 μM Sim‐H respectively. (D) Stimulation of [^3^H]‐ryanodine binding to sheep cardiac heavy SR membrane vesicles by Sim‐H (at indicated concentrations) or caffeine (10 mM) (mean and SEM; *n* = 7; *P < 0.05).

Since most ligands that interact with RyR1 also bind to RyR2, we investigated the effects of Sim‐H on the the single channel behaviour of sheep cardiac RyR2. Unexpectedly, we found that 1 μM Sim‐H (a concentration that activated RyR1) significantly decreased Po while 10 μM produced no observable effect (Figure 6A, B). The main reason for the reduction in Po was the decreased frequency of channel opening evidenced by the large increase in mean closed times with no significant change to mean open times (Figure 6C). Interestingly, at higher concentrations (10 μM), the inhibition in Po was reversed suggesting that there may be two sites of interaction: a high affinity inhibition site and a lower affinity activation site. In line with the single‐channel data, we found that [^3^H]‐ryanodine binding to sheep cardiac heavy SR membrane vesicles was inhibited by low concentrations of Sim‐H (0.5 μM) and that this effect was reversed for concentrations ≥10 μM (Figure 6D).

### Acute simvastatin perfusion alters Ca^2+^ spark characteristics in cardiac and skeletal myocytes

**Figure 7:**
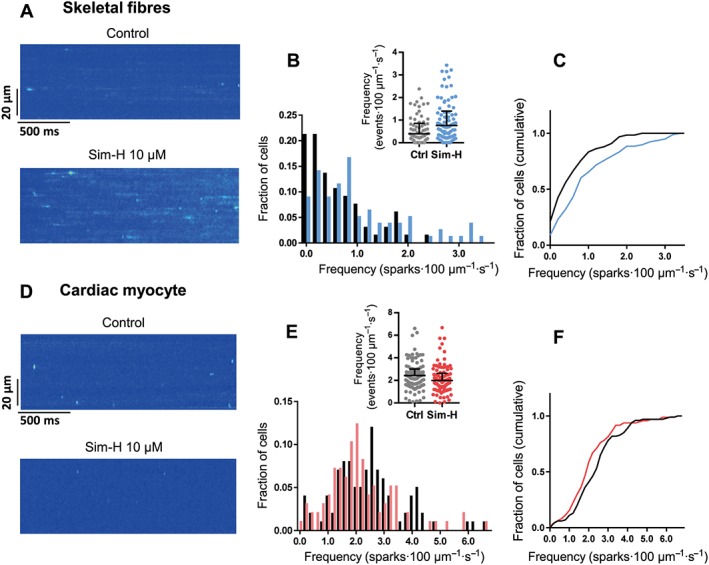
The effects of Sim‐H on spontaneous SR Ca^2+^ release in permeabilised skeletal and cardiac myocytes. (A) Representative linescan confocal images showing Ca^2+^ sparks in saponin‐permeabilised skeletal FDB fibres after 5 min perfusion with intracellular solution (Control) or intracellular solution containing 10 μM Sim‐H. (B) Frequency distribution histogram for Ca^2+^ spark frequency in FDB; inset bar graph shows raw data with median + interquartile range. Ca^2+^ spark frequency was significantly higher (*P* < 0.05) when analysed using each cell as an independent sample but was not significantly different when each rat was used as the independent sample (Mann–Whitney). (C) Cumulative frequency distribution for Ca^2+^ spark frequency in FDB. Data are from 66 control (black/grey) and 78 statin‐treated cells (blue) from *n* = 5 rats. Bin size = 0.2 sparks·100 μm^−1^·s^−1^; tickmarks on X‐axis denote bin centre for both groups. (D) Representative linescan confocal images showing Ca^2+^ spark parameters in saponin‐permeabilised cardiac ventricular myocytes perfused with intracellular solution with or without 10 μM Sim‐H for 5 min. (E) Frequency distribution histogram for Ca^2+^ spark frequency in cardiac myocytes; inset bar graph shows raw data with median + interquartile range. Ca^2+^ spark frequency was significantly lower (*P* < 0.05) when analysed using each cell as an independent sample but was not significantly different when each rat was used as the independent sample (Mann–Whitney). (F) Cumulative frequency distribution for Ca^2+^ spark frequency in cardiac myocytes. Data are from 100 control (black/grey) and 97 statin‐treated cells (red) from *n* = 5 rats. Bin size = 0.2 sparks·100 μm^−1^·s^−1^; tickmarks on X‐axis denote bin centre for both groups.

Next, we wanted to investigate whether effects of Sim‐H on RyR1 and RyR2 would be replicated in a more physiological setting. Skeletal muscle fibres and ventricular myocytes from the rat were permeabilised by saponin (to allow free access of drug to the SR membrane) and perfused acutely with 10 μM Sim‐H for 5 min. Ca^2+^ sparks, spontaneous releases of Ca^2+^ from a cluster of RyR channels, were measured in confocal linescan mode. Sim‐H appeared to exert opposing effects on Ca^2+^ spark frequency in skeletal fibres and cardiac myocytes. Representative linescan images illustrate typical Ca^2+^ spark frequency observed in skeletal fibres (Figure 7A) and cardiac myocytes (Figure 7D) in the absence and presence of Sim‐H. The shift towards higher Ca^2+^ spark frequency in FDB fibres treated with Sim‐H can be visualized in Ca^2+^ spark frequency distribution (Figure 7B) and cumulative frequency distribution (Figure 7C) plots. Similarly, the shift towards lower Ca^2+^ spark frequency in cardiac myocytes with Sim‐H treatment is shown in Figure 7E, F. There was no observed effect of Sim‐H on Ca^2+^ spark amplitude, width or duration (data not shown).

## Discussion

Our study demonstrates that simvastatin, a commonly prescribed statin, can directly modulate the gating of skeletal RyR1 and cardiac RyR2 channels incorporated into planar phospholipid bilayers. We showed that the mechanisms controlling simvastatin‐induced changes in gating are different for RyR1 and RyR2 since simvastatin increases RyR1 Po but can inhibit the opening of RyR2. Simvastatin increased the Po of RyR1 in a concentration‐dependent fashion. Simvastatin also shifted the distribution of Ca^2+^ sparks towards higher frequencies in permeabilised skeletal fibres consistent with activation of RyR1 *in situ*. The ability of simvastatin to activate RyR1 may be common to other statins and could explain why cerivastatin, a compound removed from the market due to reports of rhabdomyolysis, was shown to trigger Ca^2+^ release from isolated skeletal SR vesicles (Inoue *et al.,* 2003).

In our study, activation of RyR1 by Sim‐H was rapid and was completely reversible after wash‐out of the drug from the cytosolic chamber (Figure 1). In contrast, the addition of Sim‐H to the trans (luminal) chamber did not alter RyR1 Po (Figure 2B), suggesting that the Sim‐H binding site resides on the cytosolic side of RyR1. Lifetime analysis demonstrated that the primary mechanism driving the Sim‐H‐induced increase in RyR1 Po at low μM concentrations was the increase in frequency of channel opening (Figure 3). This is also the mechanism by which an increase in cytosolic Ca^2+^ causes activation of RyR1 (Smith *et al.,* 1986) and RyR2 channels (Ashley and Williams, 1990; Sitsapesan and Williams, 1990) suggesting that Sim‐H may be sensitizing the RyR1 channels to cytosolic Ca^2+^. This mechanism of action is also seen for other agents such as caffeine (Sitsapesan and Williams, 1990) and adenine nucleotides (Kermode *et al.,* 1998). Evidence of this mechanism can be seen in Figure 4 where the activating action of Sim‐H is abolished when cytosolic [Ca^2+^] is lowered to sub‐activating concentrations. Again, similar to caffeine, when high concentrations of Sim‐H are applied, a cytosolic Ca^2+^‐independent activation can be initiated. Our results may therefore explain why, in human permeabilised muscle fibres, application of simvastatin (10–200 μM) produces a [Ca^2+^]_i_ transient which is comparable to that of caffeine and inhibited by pretreatment of fibres with ryanodine (Sirvent *et al.,* 2005b).

It is well documented that leaky RyR1 channels are associated with skeletal myopathies such as CCD and MH (Chelu *et al.,* 2006; Guis *et al.,* 2006; Metterlein *et al.,* 2010; Hedenmalm *et al.,* 2015). In fact, patients susceptible to MH are reported to be at higher risk of developing muscular side effects including rhabdomyolysis when taking statin treatment (Metterlein *et al.,* 2010). This is unsurprising given that we have demonstrated that simvastatin increases cytosolic Ca^2+^ sensitivity of RyR1; an effect expected to potentiate any tendency for an already hyperactive population of RyR1 channels. In line with this idea, Knoblauch *et al*. (2013) reported that mice with the MH mutation, Y524S, experience an extreme hypermetabolic response to simvastatin treatment, associated with enhanced SR Ca^2+^ release from FDB fibres. The introduction of AICAR, a known RyR1 inhibitor, prevented Ca^2+^ release in both mutant and wild‐type muscle fibres. This is consistent with our findings that simvastatin causes SR Ca^2+^‐release by increasing the cytosolic Ca^2+^‐sensitivity of RyR1.

We also found that the lactone form, Sim‐L, significantly increased the Po of RyR1 (Figure 5), a result that is important for the future design of cholesterol‐lowering agents with reduced muscular side effects. While there are a variety of structurally distinct statin drugs currently available, they all possess certain common features. Type 1 statins (e.g. simvastatin, mevastatin) are natural products or derived from natural products and contain a *trans*‐decalin‐ring motif, while the more recently developed, fully synthetic, type 2 statins (e.g. atorvastatin, fluvastatin) contain a pyrrole core (Istvan, 2003). However, common to all statins is a dihydroxypentanoic acid unit which has been shown to be the crucial pharmacophore for binding to HMG‐CoA reductase (Istvan and Deisenhofer, 2001). It is well established that the statin pharmacophore may interconvert between a closed (lactone) form and an open (hydroxy acid) form. This interconversion occurs readily *in vivo* and shows a high level of pH dependence (Skottheim *et al.,* 2008). Since both Sim‐H and Sim‐L activated RyR1 in our hands (Figure 5), it was critical to ensure that the two forms of simvastatin were not interconverting during the single‐channel experiment. LC‐MS confirmed that the interconversion was too slow to be relevant on the time‐scale of our single‐channel experiments. The ability of both Sim‐L and Sim‐H to activate RyR1 suggests that the statin pharmacophore for HMG‐CoA reductase is not crucial for binding to RyR1. Thus, an attractive starting point for medicinal chemistry would be to design an inhibitor of HMG‐CoA reductase without an additional ability to activate RyR1. The number of patients becoming eligible for statin therapy has increased in recent years; in the UK, this is as a result of the lowering of the threshold 10 year cardiovascular risk for statin prescription from 20 to 10% in 2014 (NICE, 2014). However, the muscle‐related side effects are such that many patients are prescribed a lower dose of a statin drug than might otherwise be needed to achieve the desired decrease in LDL cholesterol. In some cases, patients stop taking statins altogether with implications for cardiovascular health. The development of a new class of statin molecules with reduced interaction with RyR1 may therefore particularly benefit statin users who are susceptible to myopathy.

In man, an oral dose of 40 mg simvastatin has been shown to give peak serum levels of up to 10 ng·mL^−1^ (24 nM) (Ziviani *et al.,* 2001); however, therapeutic regimes of high dose simvastatin (up to 80 mg·day^−1^) have been shown to lead to accumulation of simvastatin in tissues at levels in excess of this (e.g. 12 μM in stomach, 1 μM in spleen and testis) (Germershausen *et al.,* 1989; Rodrigues, 2010). Thus, the concentrations of statins that modulate RyR function in our experiments (≥1 μM) may be similar to those levels that accumulate inside the cells of patients taking statins. There are also several well‐established drug interactions that arise because other drugs can further increase statin plasma concentration if co‐administered. An example of such a drug is gemfibrozil, which is known to inhibit cytochrome P450, the enzyme responsible for statin metabolism, thus increasing statin plasma concentrations and increasing the occurrence of rhabdomyolysis events (Backman *et al.,* 2000; Chang *et al.,* 2004). The closed lactone form of statins is much more lipophilic than its open form counterparts; in particular, Sim‐L is around three orders of magnitude more lipophilic than Sim‐H (Serajuddin *et al.,* 1991). The potential for *in vivo* interconversion of Sim‐H to Sim‐L also increases the potential for increasing concentrations of this lipophilic form to remain in muscle tissue, despite apparently lower plasma concentrations (Skottheim *et al.,* 2008). The relatively high lipophilicity of Sim‐L would drive its accumulation in tissue and would promote higher concentrations of statin inside cells with consequences for RyR channel function. The importance of lipophilicity is supported by the finding that the relative severity of statin side effects is not directly related to efficacy of HMG‐CoA reductase inhibition. Rosuvastatin is the most potent statin in terms of reducing serum LDL cholesterol levels, but muscular related side effects are lower than with simvastatin (Jones *et al.,* 2003).

A significant finding of this work is that Sim‐H lowers the Po of RyR2 at a concentration (1 μM) that significantly activates RyR1. Higher concentrations then reverse the inhibition of RyR2 indicating that there may be a high affinity inactivation site and a lower affinity activation site on RyR2. The distribution of Ca^2+^ sparks was also shifted towards a lower frequency when isolated permeabilised cardiomyocytes were perfused with Sim‐H, consistent with inhibition of RyR2 *in situ*.

Thus, the ability of simvastatin to inhibit RyR2 channel opening could provide protection against those type of arrhythmias arising from SR Ca^2+^‐ leak. This is important since a significant proportion of statin users are already predisposed to Ca^2+^‐dependent arrhythmias and sudden cardiac death (Ko *et al.,* 2004). In future studies, it will be essential to investigate if other prescribed statins also inhibit RyR2 while activating RyR1. Our results may explain why previous reports have suggested that SR Ca^2+^ homeostasis is altered in statin‐treated skeletal muscle fibres while this is not reported for cardiac muscle (Sirvent *et al.,* 2005a).

Interestingly, simvastatin was found to reduce fast reentrant arrthymias in a rabbit model of experimental atrioventricular nodal reentrant tachycardia (Khori *et al.,* 2015). In relation to our results, it is conspicuous that statins have previously been reported to be associated with a decrease in instances of atrial fibrillation (AF) (Siu *et al.,* 2003; Young‐Xu *et al.,* 2003; Amar *et al.,* 2005) and ventricular arrhythmias in high‐risk patients (De Sutter *et al.,* 2000). A recent trial reported that statin therapy significantly reduced the occurance of supraventricular arrhythmias in patients by 29% and prevented recurrence by 33% (Biton *et al.,* 2015). A systematic review also highlighted the evidence that preoperative statin therapy can reduce the incidence of postoperative AF (Kuhn *et al.,* 2014) in patients undergoing cardiac surgery.

It should be noted that RyR2‐independent effects of statins may also contribute to their ability to protect against cardiac arrhythmia. For example, statins can alleviate the arrhythmogenic impact of ischaemia/reperfusion by restoration of myocardial perfusion through regression of atheroma (Carnicka *et al.,* 2011; Birnbaum *et al.,* 2015), increased NO bioavailability and inhibition of platelet aggregation (Tousoulis *et al.,* 2007). Ischaemia‐reperfusion has also been linked to redox modifications of RyR2, and it is possible that interaction with a statin reduces oxidative damage (Becerra *et al.,* 2016).

The effects of statins have been widely investigated, and it is likely that a number of mechanisms are responsible for their differential effects in sketetal and cardiac tissues. In particular, statins have been shown to limit mitochondrial biogenesis and impair mitochondrial function in glycolytic skeletal muscle while promoting mitochondrial biogenesis in cardiac muscle (Bouitbir *et al.,* 2012). These effects have been linked with a marked statin‐induced increase in oxidative stress in skeletal muscle compared with the relatively diminutive increase in ROS seen in cardiac muscle (Bouitbir *et al.,* 2012). This, in turn, can be explained by the greater antioxidant capacity of cardiac versus skeletal muscle (Bouitbir *et al.,* 2012; Bouitbir *et al.,* 2016).

There is no doubt that the influence of statins on arrhythmia generation may be multifaceted; however, it is tempting to speculate that inhibition of RyR2 channel opening by simvastatin is one mechanism capable of confering benefit in those patients with increased risk of sudden cardiac death by reducing the opportunity for unregulated diastolic SR Ca^2+^ leak.

In summary, we demonstrated that simvastatin can act both as an activator of RyR1 and an inhibitor of RyR2, results that were observed at the single‐channel level and confirmed by measuring Ca^2+^‐release in permeabilised isolated skeletal and cardiac cells. We suggest that these effects may underlie the tendency of simvastatin (and perhaps other statins) to cause skeletal muscle side effects but could also contribute to the reported beneficial antiarrhythmic properties of statins.

## Author contributions

R.S., E.V., C.L., S.C., S.L. and A.J.R. conceived and designed the study, E.V. and C.L. performed single‐channel experiments and analysed data. K.W. and C.L. performed and analysed [^3^H]‐ryanodine binding experiments. K.W. and A.W. isolated SR vesicles. C.L. and J.R.W. performed LC‐MS experiments, and C.L. analysed the results. S.L., Z.Y., E.S. and S.C. performed and analysed cellular Ca^2+^ spark experiments. R.S., E.V., C.L. and S.C. prepared the manuscript. All authors discussed the results and critiqued the manuscript for intellectual content.

## Conflict of interest

The authors declare no conflicts of interest.

## Declaration of transparency and scientific rigour

This Declaration acknowledges that this paper adheres to the principles for transparent reporting and scientific rigour of preclinical research recommended by funding agencies, publishers and other organisations engaged with supporting research.

## Supporting information


**Table S1** The effects of Sim‐H on lifetime parameters.
**Table S2** The effects of Sim‐H on lifetime parameters at low Ca^2 +^.
**Table S3** LC‐MS analysis of statin samples.
