# Supplementary material for: Simvastatin activates single skeletal RyR1 channels but exerts more complex regulation of the cardiac RyR2 isoform
Source: Br J Pharmacol. 2018 Feb 5;175(6):938–52. doi: 10.1111/bph.14136 (PMC5825303; doi:10.1111/bph.14136)
Supplement: Supplementary file 1 — Table S1 The effects of Sim‐H on lifetime parameters. Table S2 The effects of Sim‐H on lifetime parameters at low Ca2 +. Table S3 LC‐MS analysis of statin samples. [file BPH-175-938-s001.pdf]

Simvastatin activates single skeletal RyR1 channels but exerts more complex regulation of the cardiac RyR2 isoform

Elisa Venturi[1]*, Chris Lindsay[1,3]*, Sabine Lotteau[2], Zhaokang Yang[2], Emma Steer[2], Katja Witschas[1], Abigail D. Wilson[1], James R. Wickens[3], Angela J. Russell[1,3], Derek Steele[2], Sarah Calaghan[2], & Rebecca Sitsapesan[1]

[1] Department of Pharmacology, University of Oxford, UK
[2] School of Biomedical Sciences, University of Leeds, UK
[3] Department of Chemistry, Chemistry Research Laboratory, University of Oxford, UK

*These authors contributed equally to this work

Corresponding author. Rebecca Sitsapesan, Department of Pharmacology, University of Oxford, Mansfield Road, Oxford, OX1 3QT, UK; Tel: +441865271899; Fax: +441865271853; Email: rebecca.sitsapesan@pharm.ox.ac.uk

Supplementary Tables

Table S1. The effects of Sim-H on lifetime parameters

open times

	**10 μM Ca^{2+}**								**after 10 μM Sim-H**							
	T1 (ms)	A1 (%)	T2 (ms)	A2 (%)	T3 (ms)	A3 (%)	T4 (ms)	A4 (%)	T1 (ms)	A1 (%)	T2 (ms)	A2 (%)	T3 (ms)	A3 (%)	T4 (ms)	A4 (%)
**channel 1**	1.3	100							1	73	7	26	199	1		
**channel 2**	1.2	100							1	83	5.2	16	98	1		
**channel 3**	1	94	5	6					2	63	24	27	304	10		
**channel 4**	1.2	100							1.3	61	9.5	30	91	9		
**channel 5**	0.59	100							0.70	81	2.8	19				

closed times

	**10 μM Ca^{2+}**								**after 10 μM Sim-H**							
	T1 (ms)	A1 (%)	T2 (ms)	A2 (%)	T3 (ms)	A3 (%)	T4 (ms)	A4 (%)	T1 (ms)	A1 (%)	T2 (ms)	A2 (%)	T3 (ms)	A3 (%)	T4 (ms)	A4 (%)
**channel 1**	1	30	7	33	149	31	7075	6	2	69	29	28	1765	3		
**channel 2**	2.5	32	23	44	123	23	3276	1	2.4	59	20	35	167	6		
**channel 3**	2	58	18	29	200	11	11897	2	1.5	76	15	22	557	2		
**channel 4**	3	32	45	37	457	23	4771	8	2	67	28	29	1094	4		
**channel 5**	1.3	20	5	37	36	33			1	67	7.2	33				

Table S1. The effects of Sim-H on lifetime parameters Time constants (T1, T2, T3, T4) and percentage areas (A1, A2, A3, A4) obtained from maximum likelihood fitting of pdfs to open and

close lifetime distributions of 5 independent single RyR1 channels in the presence and absence of 10 µM Sim-H are shown .

Table S2. The effects of Sim-H on lifetime parameters at low Ca^{2+}

open times

	**1 nM Ca^{2+} and 50 µM Sim-H**							
	T1 (ms)	A1 (%)	T2 (ms)	A2 (%)	T3 (ms)	A3 (%)	T4 (ms)	A4 (%)
**channel 1**	2.2	23	14.3	44	69.5	33		
**channel 2**	1.7	32	8.8	34	76.2	33		
**channel 3**	1.5	41	7.3	29	59	29		
**channel 4**	1.0	57	6.0	35	55.0	8		
**channel 5**	1.4	56	6.2	30	53.4	14		

Table S2. The effects of Sim-H on lifetime parameters at low Ca^{2+} Time constants (T1, T2, T3, T4) and percentage areas (A1, A2, A3, A4) obtained from maximum likelihood fitting of pdfs to open lifetime distributions of 5 independent single RyR1 channels in the presence 1 nM Ca^{2+} and 50 µM Sim-H are shown.

Table S3. LC-MS analysis of statin samples

Retention [min]	Flow [ml/min]	% Acetonitrile	% Water + 0.1 % Formic acid
0	0.4	5	95
0.5	0.4	5	95
3.5	0.4	99	1
4	0.4	99	1
4.1	0.4	5	95
5	0.4	5	95

Table S3. LC-MS analysis of statin samples. A mixed gradient of eluent was used as described in the table, with the percentage of acetonitrile/water/formic acid being varied with retention time as indicated.
